# Lifestyle changes during the first wave of the COVID-19 pandemic: a cross-sectional survey in the Netherlands

**DOI:** 10.1186/s12889-021-11264-z

**Published:** 2021-06-25

**Authors:** Esther T. van der Werf, Martine Busch, Miek C. Jong, H. J. Rogier Hoenders

**Affiliations:** 1 Louis Bolk Institute, Bunnik, The Netherlands; 2 Dutch Consortium for Integrative Medicine and Health (CIZG), Utrecht, The Netherlands; 3 Van Praag Institute, Utrecht, The Netherlands; 4 National Research Center in Complementary and Alternative Medicine (NAFKAM), Department of Community Medicine, UiT The Arctic University of Norway, Tromsø, Norway; 5 Mid Sweden University, Department of Health Sciences, Sundsvall, Sweden; 6 Center for Integrative Psychiatry (CIP), Lentis Mental Health Institution, Groningen, the Netherlands

## Abstract

**Background:**

During the Covid-19 pandemic the Dutch government implemented its so-called ‘intelligent lockdown’ in which people were urged to leave their homes as little as possible and work from home. This life changing event may have caused changes in lifestyle behaviour, an important factor in the onset and course of diseases. The overarching aim of this study is to determine life-style related changes during the first wave of the COVID-19 pandemic among a representative sample of the adult population in the Netherlands.

**Methods:**

Life-style related changes were studied among a random representative sample of the adult population in the Netherlands using an online survey conducted from 22 to 27 May 2020. Differences in COVID-19-related lifestyle changes between Complementary and Alternative Medicine (CAM) users and non-CAM users were determined. The survey included a modified version of the I-CAM-Q and 26 questions on lifestyle related measures, anxiety, and need for support to maintain lifestyle changes.

**Results:**

1004 respondents were included in the study, aged between 18 and 88 years (50.7% females). Changes to a healthier lifestyle were observed in 19.3% of the population, mainly due to a change in diet habits, physical activity and relaxation, of whom 56.2% reported to be motivated to maintain this behaviour change in a post-COVID-19 era. Fewer respondents (12.3%) changed into an unhealthier lifestyle. Multivariable logistic regression analyses revealed that changing into a healthier lifestyle was positively associated with the variables ‘Worried/Anxious getting COVID-19’ (OR: 1.56, 95% C.I. 1.26–1.93), ‘CAM use’ (OR: 2.04, 95% C.I. 1.38–3.02) and ‘stress in relation to financial situation’ (OR: 1.89, 95% C.I. 1.30–2.74). ‘Age’ (OR 18–25: 1.00, OR 25–40: 0.55, 95% C.I. 0.31–0.96, OR 40–55:0.50 95% C.I. 0.28–0.87 OR 55+: 0.1095% C.I. 0.10–0.33), ‘stress in relation to health’ (OR: 2.52, 95% C.I. 1.64–3.86) and ‘stress in relation to the balance work and home’ (OR: 1.69, 95% C.I. 1.11–2.57) were found predicting the change into an unhealthier direction.

**Conclusion:**

These findings suggest that the coronavirus crisis resulted in a healthier lifestyle in one part and, to a lesser extent, in an unhealthier lifestyle in another part of the Dutch population. Further studies are warranted to see whether this behavioural change is maintained over time, and how different lifestyle factors can affect the susceptibility for and the course of COVID-19.

**Supplementary Information:**

The online version contains supplementary material available at 10.1186/s12889-021-11264-z.

## Background

The rapid spread of COVID-19 to nearly all parts of the world has posed enormous health, economic, environmental and social challenges worldwide. In the absence of effective drugs or vaccines, social distancing, surgical masks, washing hands and other preventive measures are presented as the only ways to fight the (spread of the) virus. Lockdown is among one of the options suggested by WHO to reduce spread of the virus. Although underreported, preventative strategies such as a healthy lifestyle seem important alternative avenues to fight (the spread of) COVID-19. From a public health perspective, these strategies are very important to consider. Between February 2020 and 1st of June 2021 1.651.780 positive cases and 17,632 deaths has been registered in The Netherlands [1]. As a response to COVID-19, many countries are using a combination of containment and mitigation activities with the intention of delaying major surges of patients and levelling the demand for hospital beds, while protecting the most vulnerable from infection, including elderly people and those with comorbidities [2]. In the Netherlands, a so-called “intelligent lockdown” was enforced on 15th of March 2020, with easing of restrictions per 1st of July 2020 [3]. With the intelligent lockdown, the Dutch Government appealed to the responsibility and self-discipline of citizens to practice 1.5 m social distance, and to maintain home isolation when showing COVID-19-related symptoms. Over the course of several weeks in March and April 2020, additional measures were taken to restrict the further spread of the coronavirus in the Netherlands. These measures included closure of schools, restaurants, certain beaches and parks, and prohibition of spontaneous group gatherings in public spaces.

Due to this intelligent lockdown, a sudden and radical change occurred in the lives and habits of the Dutch population. Life experiences that may greatly influence an individual’s daily routine are referred to as life changing events [4], defined as “those occurrences, including social, psychological and environmental, which require an adjustment or effect a change in an individual’s pattern of living.” Life changing events may influence lifestyle behaviours for better or worse [5, 6]. For instance, Engberg et al. showed that transition to university, having a child, remarriage and mass urban disasters were associated with decreased physical activity levels, while retirement was associated with increased physical activity [7]. Stressful life events have been correlated with excessive alcohol consumption and alcohol dependence and emotional eating [8].

Maintaining a healthy nutrition status and level of certain exercise is crucial, especially in a period when the immune system might need to fight back. In fact, subjects with (severe) obesity (BMI ≥ 30 kg/m2) are one of the groups with a higher risk for COVID-19 complications [9, 10]. Therefore, losing weight may be one of the strategies to lower the risk of severe illness from COVID-19. Worldwide, authorities and healthcare professional’s recommendations on how to stay healthy during the COVID-19 pandemic, besides taking appropriate hygiene measures, are related to healthy life-style measures such as assuring sufficient sleep, eat plenty of fresh fruits and vegetables, reduce stress and social isolation and stay active [11, 12].

The COVID-19 pandemic might motivate people to make healthier choices and adopt a healthier lifestyle. Conversely, COVID-19 control measures such as social distancing and compulsory home isolation can be expected to increase sedentary behaviour and might cause an unhealthy eating and sleeping pattern. For example, the interruption of the daily (work) routine caused by the staying at home (which includes digital-education, working from home, and limitation of outdoors and in-gym physical activity) could result in boredom, which in turn is associated with a greater energy intake [13]. In addition, hearing or reading continuously about the COVID-19 pandemic and its possible impact from media can be stressful. Stress leads individuals toward overeating, especially ‘comfort foods’ or inactivity [14]. For future actions it is important to determine the lifestyle changes taken during this COVID-19 pandemic, and what support will be needed to (dis) continue this health behaviour in a post-COVID-19 era.

Previous studies show that Complementary and Alternative Medicine (CAM) users have on average a healthier lifestyle behaviour than non-CAM users, and overall a stronger focus on wellness [15–18]. In general, CAM is defined as a group of diverse medical and health care symptoms, practices and products that are not generally considered part of conventional medicine [19]. Nahin et al. found based on a survey among the US population that engaging in leisure-time physical activity, having consumed alcohol in one’s life but not being a current heavy drinker, and being a former smoker are independently associated with the use of CAM [16]. Interestingly, reported significantly better health status and healthier behaviours overall (higher rates of physical activity and lower rates of obesity) seems more prominent in adults using CAM for health promotion than those who use CAM as treatment [15]. The relation between CAM use and lifestyle needs further investigation in various populations.

The overarching aim of this reported study is to investigate life-style related changes during the first wave of the COVID-19 pandemic among a representative sample of the adult population in the Netherlands. Within this aim the following objectives has been framed: i) To determine life-style related changes (healtier/unhealthier) during the first wave of the COVID-19 pandemic; (ii) To identify the (sociodemographic) factors independently associated with changes into lifestyle (healthy/unhealthy); (iii) To explore possible differences in COVID-19-related lifestyle changes between CAM users and non-CAM users, and (iv) To determine the intention to continue lifestyle changes and the required support.

## Methods

An international cross-sectional survey on CAM use and self-care strategies for prevention and treatment of COVID-19 related symptoms was carried out in Norway, Sweden and the Netherlands in spring 2020. The results of this international survey will be published elsewhere. This online survey consisted of a modified version of the International Questionnaire to Measure Use of Complementary and Alternative Medicine (I-CAM-Q) [20], and a country specific part on lifestyle for The Netherlands (it is the latter on which this paper reports). The modified I-CAM-Q consisted of four parts, and all parts related to CAM use during the past three COVID-19 pandemic months as did the Dutch part on lifestyle.

The modified I-CAM-Q included questions about visits to conventional and unconventional health care providers, self-management strategies such as use of natural remedies and self-help techniques such as mindfulness used within the last three months. The questions regarding specific therapies were adapted to The Netherlands (supplementary material).

The country specific part for the Netherlands included 26 questions divided into three sections on 1) current lifestyle related measures (alcohol use, smoking, daily consumption of certain foods, exercise, sleep, stress and meaning and purpose/spirituality), 2) lifestyle related changes since the COVID-19 outbreak and anxiety (section 1 and 2: 20 questions) and 3) intention to continue lifestyle changes and need for support (6 questions). For this study, we included six aspects of lifestyle with established effects on physical and mental health: nutrition, exercise, sleep, addiction, relaxation and meaning and purpose/spirituality.

In the Netherlands, an online survey was performed between May 22 and May 27, 2020 in collaboration with Ipsos Netherlands. An internal Ipsos tool (ISS) has been used to gather the respondents. The respondents registered into the IIS panel have shared their baseline information such as age,gender, region, and more specific information on education, income and work. From the panel of 45,000 Dutch residents, a representative sample (based on the baseline parameters) was invited to complete the questionnaire until 1000 responses were received (limit set due to costs). Individuals who were reached and refused participation (*n* = 3607) were considered non-respondents, leading to a response rate of 22%. The final sample contained 1004 individuals.

Taking into account multiple response biases, the questionnaire was designed as followed: 1) answer options were randomized. Meaning every participant will see the same answer options, but in different order, preventing primacy bias (to decrease the amount of times one answer can be chosen which might lead to survey results being too unfairly weighted towards one option), and 2) questions were formulated in a neutral way when asked about education level, salary, age and gender to prevent prestige/stereotype bias as much as possible. Respondents received a personal link (password/username) to prevent filling in the questionnaire more than one time or any self-selection bias would happen.

Demographic characteristics collected were gender, age, municipality of residence and county, income, and level of education. Income was classified as high (Euro 75,000 >), middle (Euro 25,000 – 74,999) and low income (< Euro 24,999). Education was classified as higher education ((applied) university/ post-doctoral level), secondary education (middle and higher secondary education) and lower education (no school/primary school only/lower secondary education).

All data was anonymously collected and reported. The anonymous nature of the web-survey did not allow to trace in any way sensitive personal data. The study protocol was reviewed by the Medical Ethical Reviewing Committee of Wageningen University. They decided that this study did not fall within the remit of the Dutch Medical Research Involving Human Subjects Act (WMO), and therefore was exempt from further medical ethical review. Informed consent was obtained from all participants and all patients agreed their data to be used for scientific publication. GDPR guidelines were taken into account [21]. Once completed, each questionnaire was transmitted to the survey platform, and the final database was downloaded.

The current paper reports on the country specific part of the survey using data of the I-CAM-Q, only to categorize users and non-users of CAM. Here, CAM use is defined as all treatments and (self) care strategies that are used in addition or as an alternative to the usual (regular) care of e.g. general practitioner, specialist, dietician, physiotherapist or nurse in the past 3 months.

### Statistical analyses

Descriptive statistics like measures of central tendencies, frequencies and proportions were used to evaluate the responses. Data are represented as number and/or percentage for categorical variables or mean and standard deviation for continuous variables. Pearson’s Chi-square test and ANOVA tests were performed to identify differences in socio-demographics (age, education level, household income), as well as to identify differences in lifestyle/lifestyle changes between users and non-users of CAM.

Univariable and multivariable logistic regression was used to identify the (sociodemographic) factors independently associated with changes in lifestyle (healthy/unhealthy). Outcomes on changes in lifestyle questions were dichotomized. Change in lifestyle due to corona crisis: answer categories: Yes, I live healthier, Yes, I live unhealthier and No. Multivariable models were derived through several iterations using backward stepwise logistic regression, including all variables that were statistically significant in the univariable analyses. The authors controlled for age, gender and education in these models.

Statistics were carried out using Statistical Package for Social Sciences (SPSS) v. 26.0. Results were statistically significant for *p* value < 0.05.

## Results

A total of 1013 individuals completed the online questionnaire, and, after validation of the data, 1004 respondents (age 18–88 years) were included in the study. As shown in Table 1, most respondents were between 50 to 69 years of age (37.5%), and female respondents represented 50.7% of the population sample. Respondents were distributed across the 12 provinces, with 27.3% from the northern regions of the Netherlands, 27.6% from the central regions of the Netherlands and 45.1% from the southern regions of the Netherlands. Of all respondents, 46.5% resided in urban zones, 23.8% in sub-urban zones and 24.9% in rural/sub-rural zones. Married respondents living with or without children accounted for the majority of sample distribution, making up to 63.3% of responses followed by individuals living alone without children (24.8%). Half of the respondents (49.9%) had a higher education status and 49.7% of respondents was categorized to have a middle income.

**Table 1:** Baseline and socio-demographic characteristics

	Total population (*n* = 1004)
	n (%)
Age category	
18–30	176 (17.5)
31–50	316 (31.5)
51–65	374 (37.3)
65+	138 (13.7)
Gender	
Female	509 (50.7)
Male	495 (49.3)
Region (The Netherlands)	
Northern regions	274 (27.3)
Central regions	277 (27.6)
Southern regions	453 (45.1)
Living environment	
Urban	467 (46.5)
Sub-urban	239 (23.8)
Rural/sub-rural	298 (24.9)
Living situation	
Married/living together (without children)	386 (38.4)
Married/living together (with children)	250 (24.9)
Living alone without children	249 (24.8)
Living alone with children	33 (3.3)
Living with (grand)parents/family	73 (7.3)
Student accommodation	13 (1.3)
Education*	
Lower education	167 (16.6)
Secondary education	336 (33.5)
Higher education	501 (49.9)
Yearly income (per household)**	
Lower income	150 (14.9)
Middle income	499 (49.7)
Higher income	146 (14.5)
Prefer not to say	209 (20.8)
Lifestyle	
Smoking (yes)	153 (15.2)
Alcohol use (yes)	637 (63.4)
	**Mean (sd)**
Age	48.9 (17.3)
Cigarettes/day	12.3 (7.5)
Glasses alcohol/week	3.7 (6.1)
Days with 30 min. Exercise	4.3 (2.3)
Sleep quality (0 (low)-10 (high))	6.9 (1.7)

* higher education ((applied) university/ post-doctoral level), secondary education (middle and higher secondary education) and lower education (no school/primary school only/lower secondary education)

** higher (> Euro 75.000), middle (Euro 25.000–75.000) and lower income (< Euro 25.000)

### Lifestyle changes during the COVID-19 pandemic

Although the majority of the surveyed population reported no significant change in their daily habits or intake of food/snacks since the COVID-19 outbreak in the Netherlands, we found substantial lifestyle changes in a considerable part of the population, both for the better and the worse (see Table 2). 14.0% of all respondents reported a decrease in sleeping hours, while 13.0% reported an increase. One fifth (20.0%) of the respondents reported to snack more than before the COVID-19 pandemic, and 7.7% snacked less. Intake of vegetables increased in 11.7% whereas it decreased in 1.7%.

**Table 2:** Lifestyle related changes since Covid-19 outbreak in The Netherlands

	Total population (*n* = 1004)			
	n (%)			
	More	Same	Less	Do not know
Alcohol use	82 (8.2)	694 (69.1)	158 (15.7)	70 (7.0)
Smoking	37 (3.7)	595 (59.3)	83 (8.3)	289 (28.8)
Exercise	228 (22.7)	503 (50.1)	245 (24.4)	28 (2.8)
Hours of sleep	131 (13.0)	714 (71.1)	141 (14.0)	18 (1.8)
Intake of fruit	136 (13.5)	806 (80.3)	50 (5.0)	12 (1.2)
Intake of vegetables	117 (11.7)	862 (85.9)	17 (1.7)	8 (0.8)
Snacks cookies/crisp, cake etc.)	201 (20.0)	602 (60.0)	77 (7.7)	23 (2.3)
More aware of food habits	165 (16.4)	757 (75.4)	55 (5.5)	27 (2.7)
Stress in relation to:				
Work	206 (20.5)	283 (28.2)	117 (11.7)	398 (39.6)
Health	223 (22.2)	525 (52.3)	70 (7.0)	186 (18.5)
Balance work/ childcare	108 (10.8)	247 (24.6)	69 (6.9)	580 (57.8)
Care for family	101 (10.1)	237 (23.6)	43 (4.3)	623 (62.1)
Income/financial situation	187 (18.6)	450 (44.8)	81 (8.1)	286 (28.5)
Future perspective	298 (29.7)	437 (43.5)	60 (6.0)	209 (20.8)

Table 2 shows that the majority did not know whether their stress levels had changed in relation to ‘the balance between work and childcare’ and ‘care for their family’, respectively 57.8 and 62.1%. 52.3% of the respondents indicated no change in stress related to their own health, but nearly a quarter (22.2%) did perceive more health-related stress or future perspective related stress (27.7%).

As shown in Tables 3, 80% of the respondents reported that in general they were happy with their current lifestyle. 12.2% of the total population reported an unhealthier lifestyle since the outbreak of the COVID-19 pandemic, whereas 19.3%, (*n* = 194) indicated that the COVID-19 pandemic positively influenced their lifestyle (Table 3). The 194 respondents reported a healthier lifestyle due to a higher intake of fruit and vegetables (54.6%), exercise (63.4%), and relaxation (46.4%). Only a small proportion of the participants reported to live healthier due to a change in meaning of life aspects/spirituality (6.2%) (Table 3).

**Table 3:** Lifestyle and COVID-19

	Total population (*n* = 1004)		
	n (%)		
	Yes	No	Do not know
In general happy with lifestyle	803 (80.0)	175 (17.4)	26 (2.6)
Changes in lifestyle could influence getting infected by the coronavirus	355 (35.4)	484 (48.2)	165 (16.4)
Changes in lifestyle could influence the symptoms of the coronavirus once infected	450 (44.8)	357 (35.6)	197 (19.6)
	**Yes, I live healthier**	**Yes, I live unhealthier**	**No**
Corona crisis influences lifestyle	194 (19.3)	123 (12.3)	687 (68.4)
Healthier lifestyle due to (*n* = 194)			
- more fruit and vegetables (yes)	106 (54.6)		
- more exercise (yes)	123 (63.4)		
- more relaxation (yes)	90 (46.4)		
- more spirituality (yes)	47 (6.2)		

Remarkably, the number of respondents that thought that lifestyle changes can influence the natural history (symptoms) of COVID-19 once infected, was higher than the number of respondents that thought lifestyle changes can influence the risk of getting infected (Table 3). Nearly halve of respondents (48.2%) did not think that a change in their lifestyle could decrease their risk of getting infected by the corona virus (Table 3).

### Factors independently associated with changes into lifestyle (healthy/unhealthy)

Table 4 shows the univariable statistically significant associated variables with a change to healthy- or unhealthy lifestyle that are entered into the multivariable analyses to come to the final models (*P* < 0.05). Based on univariable analyses, no statistically significant associations with a change to a healthy lifestyle were found with regards to age, gender, residential region, smoking, alcohol use, stress in relation to work and stress in relation to future perspectives. With regard to a change to an unhealthy lifestyle no statistically significant associations were found for gender, income level, living region, smoking, alcohol use, use of CAM and anxiety for getting infected their selves with Covid-19.

**Table 4:** Univariable- and multivariable logistic regression analyses- final models change to healthy/unhealthy lifestyle (*n* = 1004)

	Change into healthier lifestyle		Change into unhealthier lifestyle	
	Univariate significant variables (*P* < 0.05)	Final multivariable model^#^ (AOC: 0.66 (0.63–0.71))	Univariate significant variables (*P* < 0.05)	Final multivariable model^#^ (AOC: 0.56 (0.50–0.62))
	OR (95% C.I.)	OR (95% C.I.)	OR (95% C.I.)	OR (95% C.I.)
Age category				
18–24 years			1.00	**1.00**
24–39 years			0.47 (0.27–0.82)	**0.55 (0.31–0.96)**
40–54 years			0.46 (0.27–0.80)	**0.50 (0.28–0.87)**
55+ years			0.16 (0.10–0.29)	**0.10 (0.10–0.33)**
Income category*				
Lower	0.61 (0.39–0.94)			
Middle	1.01 (0.63–1.82)			
Higher	0.77 (0.47–1.28)			
Education level**				
Lower education			1.00	
Secondary education			2.79 (1.33–5.84)	
Higher education			2.76 (1.34–5.66)	
Worried/Anxious to get infected with SARS-coV-2	1.63 (1.32–2.03)	**1.56 (1.26–1.93)**		
Worried/Anxious a close relative/friend to get infected with SARS-coV-2	1.48 (1.18–1.45)		1.37 (1.05–1.79)	
CAM use	2.29 (1.56–3.37)	**2.04 (1.38–3.02)**		
Stress in relation to:				
Work			2.08 (1.37–3.14)	
Health	1.95 (1.38–2.76)		3.18 (2.14–4.70)	**2.52 (1.64–3.86)**
Balance work/life	1.52 (1.09–2.11)		2.35 (1.80–3.45)	**1.69 (1.11–2.57)**
Care for family	1.90 (1.20–3.01)		2.06 (1.22–3.48)	
Financial situation	2.10 (1.46–3.02)	**1.89 (1.30–2.74)**	2.20 (1.44–3.35)	
Future			2.08 (1.37–3.14)	

* higher education ((applied) university/post-doctoral level), secondary education (middle and higher secondary education) and lower education (no school/primary school only/lower secondary education);** higher (> Euro 75.000), middle (Euro 25.000–75.000) and lower income (< Euro 25.000); ^#^ Adjusted for age and gender

The final multivariate models (Table 4) included 1004/1004 (100%) of the respondents of the survey. Three predictors were strongly associated with changing into a healthy lifestyle: Worried/Anxious getting infected with SARS-coV-2 (OR: 1.56, 95% C.I. 1.26–1.93), CAM use (OR: 2.04, 95% C.I. 1.38–3.02) and stress in relation to financial situation (OR: 1.89, 95% C.I. 1.30–2.74). Together these gave an AUROC of 0.66 (95% CI = 0.63 to 0.71). Similarly, three predictors were strongly associated with changing into an unhealthy lifestyle: Age (OR 18–25: 1.00, OR 25–40: 0.55, 95% C.I. 0.31–0.96, OR 40–55:0.50 95% C.I. 0.28–0.87 OR 55+: 0.1095% C.I. 0.10–0.33), stress in relation to health (OR: 2.52, 95% C.I. 1.64–3.86) and stress in relation to the balance work and home (OR: 1.69, 95% C.I. 1.11–2.57). Together these gave an AUROC of 0.56 (95% C.I. 0.50–0.62)).

#### Differences in COVID-19-related lifestyle changes between CAM users and non-CAM users

Our multivariable model shows that CAM use is an important predictor of changing to a healthier lifestyle during the first wave of the COVID-19 pandemic and is not statistically significant associated with a change to an unhealthy lifestyle. More than two third (68%) of the respondents indicated use of CAM in the past 3 months. 13.3% of all respondents consulted a CAM practitioner (medical doctor or other (non) healthcare professional specialized in CAM, 59.4% used (CAM) supplements (e.g. vitamins/minerals, herbs, and/or dietary supplements) and 30% indicated to make use of (CAM) self-help techniques ((e.g. breathing exercises, yoga) (Table 5).

**Table 5:** Differences in COVID-19-related lifestyle changes between CAM users and non-CAM users

	Total Population (*n* = 1004)			
Use of CAM (yes)	683 (68.0)			
- Consulting CAM health care professional	133 (13.3)			
- Use of CAM supplements	596 (59.4)			
- use of (CAM) self-help techniques	301 (30.0)			
	**CAM Users (*n*** **= 683)**		**Non CAM users** **(*n*** **= 321)**	
	**Mean (sd)**		**Mean (sd)**	
Age	48.6 (17.2)		49.7 (16.5)	
Cigarettes/day	12.1 (6.8)		12.9 (9.3)	
Glasses alcohol/week	3.7 (6.2)		3.9 (5.6)	
Days with 30 min. Exercise	4.3 (2.3)		4.1 (2.4)	
	**n (%)**		**n (%)**	
Lifestyle could influence getting infected by COVID-19				
Yes	260 (38.1)		85 (26.7)	
No	82 (46.3)		174 (54.3)	
Do not know	106 (15.6)		60 (18.9)	
Lifestyle could influence the symptoms of COVID-19 once infected				
Yes	316 (46.3)		129 (40.3)	
No	234 (34.4)		125 (39.1)	
Do not know	131 (19.3)		66 (20.6)	
Corona crisis influences lifestyle	***n*** **= 571**		***n*** **= 243**	
In general happy with lifestyle (yes)	500 (87.7)		204 (84.0)	
Live unhealthier *(yes)*	102 (13.4)		21 (8.6)	
Live healthier *(yes)*	162 (21.3)		32 (13.2)	
	**not at all worried** **- neutral**	**worried -very much worried**	**not at all worried** **- neutral**	**worried -very much worried**
	**n (%)**		**n (%)**	
Anxious/worried to get infected with COVID-19	539 (79.0)	143 (21.0)	278 (86.8)*	42 (13.2)

*statistically significant different between CAM users and non-CAM users at *P* < 0.05

No statistically significant differences were found between non-CAM and CAM users with regards to mean age, residential region, marital status, education and yearly income. Lifestyle related behaviour measures as smoking, alcohol use and daily exercise were similarly distributed between the two groups. The younger aged (age < 30) and the elderly (age 65+) did make less use of CAM then those aged between 31 and 64 years old, as did men (male non-CAM users: 61.7%).

As shown in Tables 5, 87.7 and 84.0% of the CAM users and non-CAM users respectively reported that in general they were happy with their current lifestyle. The proportion CAM users that changed into a healthier lifestyle influenced by the COVID-19 pandemic is bigger than the proportion of non-CAM users.

More than one third of the CAM users indicated to think changes in lifestyle could change their risk of getting infected with SARS-coV2 (38.1%), and 46.3% did also think that changing their lifestyle could influence the course of the illness once infected, compared to 40.3% of the non-CAM users and 44.8% of all participants. CAM users were statistically significant less anxious/worried to get infected with COVID-19 than non-CAM users.

In general, CAM users perceived more often an increase in stress than non-CAM users. Rather large differences were found between more stress in the previous three months in relation to work (CAM users: 23.1%, Non-CAM users: 12.3%, *P* < 0.001), health (CAM users: 25.9%, Non-CAM users: 10.7%, *P* < 0.001), balance work/childcare (CAM users: 12.0%, Non-CAM users: 7.0%, *P* = 0.012)), financial situation (CAM users: 21.2%, Non-CAM users:10.7%, *P* < 0.001)) and future perspective (CAM users: 33.8, Non-CAM users:16.9%, *P* < 0.001)).

In the 3 months ahead of the survey, CAM users were more aware of their own diet habits than non-CAM users (CAM users: 18.8%, Non-CAM users: 9.1%, *P* < 0.05).

### Intention to continue lifestyle changes and the required support

This study provides information that may be relevant to policy makers, health insurances and research funding organizations to guide future decisions on lifestyle and COVID-19.

Table 6 shows that in general, more than halve of the 194 respondents who reported a positive change in their lifestyle since the start of the COVID-19 pandemic indicated the wish to continue their changes through healthy food (56.2%) and exercise (54.6%). Of the pre-defined options: 1) healthy choices at work/school (food, drinks, exercise during breaks e.g., yoga, tai chi, mindfulness) 2) free choice and reimbursement of any treatment in relation to CAM and Lifestyle; 3) support from GP/Health centre/Community care; 4) online advice and support, and 5) affordable and easilyaccessible healthy food, 55.8% of respondents declared needing none of these.

**Table 6:** Continuation of change in a post Covid-19 era and needed support

	Subgroup that changed into healthier lifestyle (*n*=194)*	
	n (%)	
Wish to continue of change:	**Yes**	**No**
Healthy food	109 (56.2)	85 (43.8)
Exercise	106 (54.6)	88 45.4)
Sleep	68 (35.1)	126 (64.9)
Stress	49 (25.3)	145 (74.7)
Spirituality	40 (20.6)	154 (79.4)
Kick off addiction (alcohol, smoking, drugs, sex, sugar, Netflix/gaming)	18 (9.3)	176 (17.5)
	**Total (*n*=1004)**	
	n (%)	
Need for (pre-defined) support options:	**Yes**	**No**
Health choices at work/school (food, drinks, exercise during breaks (yoga, tai chi, mindfulness, music)	172 (17.1)	832 (82.9)
Free choice and reimbursement of any treatment in relation to CAM and lifestyle	161 (16.0)	843 (84.0)
Support from GP/health center/ Community care	120 (12.0)	884 (88.0)
Online advice and support	92 (9.2)	912 (90.8)
None of these	560 (55.8)	444 (44.2)

* includes respondents only who indicated that the corona crisis made their lifestyle healthier

However, affordable and easily accessible healthy food was perceived as helpful by one third of the respondents (34.7%), followed by healthy choices at work/school and free choice and reimbursement of CAM and lifestyle treatments with respectively 17.2 and 16.0%.

Statistically significant more CAM users reported a desire to continue more activities regarding meaning of life/ spirituality/ (CAM users: 27.4%, Non-CAM users: 10.8%, *P* = 0.03) in a post Covid-19 era.

## Discussion

This population-based study is a snapshot of the health related lifestyle changes of Dutch residents during the first wave of the COVID-19 pandemic which included nine weeks of Intelligent lockdown as declared by the Dutch Government. Our study seems to indicate that one fifth of the Dutch residents changed their lifestyle into a healthier one and that this was mainly due to healthier food habits, more exercise and more relaxation. More than half of these respondents reported to be motivated to maintain this behaviour change in a post-COVID-19 era. Around 10% of the total study population, on the other hand, admitted to have started living unhealthier due to the corona crisis. 35% of respondents thought that a lifestyle change could change their risk of getting infected by the corona virus and nearly half of the total group thought this change could influence the course of the illness once infected.” Our study also shows that CAM use is an important predictor of changing to a healthier lifestyle during the first wave of the COVID-19 pandemic. The use of CAM and healthy lifestyle has been associated previously [15–18], and our results confirm this positive association.

Regardless of the time and context within one decides to eat better, exercise more, or be less stressed, it can be hard to make a lifestyle change, and even harder to make it a habit [22]. Life changing events might provide a unique opportunity to live healthier and to continue these changes [23]. Since the outbreak of the novel coronavirus disease (COVID-19) in China, the world is in the grip of a coronavirus pandemic, a unique crisis with disastrous health, societal and economic effects worldwide [24]. The Corona crisis is said to be the biggest crises since World War III in the Netherlands and is expected to change the way we think and live at individual and societal levels.

A large part of non-communicable diseases is caused by unhealthy behaviour [20, 25, 26]. Addressing modifiable risks such as tobacco use, physical inactivity, unhealthy diet and harmful use of alcohol are among most effective interventions to keep people healthy and productive, reducing the individual, societal and economic impact and suffering caused by non-communicable diseases [20, 25, 26]. Nearly 20% of our respondents indicated that the COVID-19 pandemic positively influenced their lifestyle. This is a positive finding from a public health perspective, in which the importance of a healthier lifestyle to prevent chronic and non-communicable diseases is emphasized. A comparable percentage among a representative sample of the general population of Italy surveyed in the first months of 2020 was found to change to a healthier lifestyle. The survey in Italy further revealed that most of the Italian respondents declared not to have changed its habits (46.1%) (compared to 68% of our respondents), while 37.2% (compared to 12% of our respondents) felt to have made them worse [27]. This latter difference might be due to the difference in lockdown, with a stricter one in Italy.

Although healthy lifestyles offer a number of health benefits, non- adherence to recommended lifestyle changes remains a frequent and difficult obstacle to realizing these benefits [28, 29]. It is therefore promising that of this representative sample of the Dutch population, more than half who changed into a healthier lifestyle indicates to be willing to maintain to these new habits. A US poll has found that as many as 80% of American adults will try to practice self-care more regularly once the COVID-19 pandemic is over [30]. The prospect of improving health and reducing illness through changes in living habits rather than through curative healthcare, is attractive from the perspective of public health and on economic grounds.

Our final multivariable model for changing into a healthy lifestyle showed positive associations with: (i) anxiety to get infected with SARs-coV-2; (ii) the use of CAM; and (iii) stress with regards to financial situation. Taylor et al. (2020) recently developed the COVID stress Scales (CSS) and identified five factors of stress and anxiety symptoms relating to the coronavirus in two large samples in Canada and the United States including ‘danger and contamination’ and ‘fears about economic consequences’. Two predictive factors (anxiety to get infected with SARs-coV-2 and stress with regards to financial situation) we found to be positively associated with a change into a healthy lifestyle. Previously, Anderson et al. showed that occurrence of life events and subsequent effects, can contribute to health promoted behaviour despite the potential worries, poor health and diseases which may also be associated with them [23].

Analyses of data from the National Health Interview Study (NHIS) found that CAM users were more likely to use exercise and less likely to be obese than those who did not use CAM [15, 16]. Associations of CAM with exercise [15, 16, 31, 32], higher vegetable intake [31–33], lower fat or lipid intake [31–33], and smoking cessation or decreased smoking [16, 31, 34] have been reported previously. These studies, like ours, show a commitment to overall wellbeing that spans both lifestyle and CAM use and hypothesise that CAM therapies may even be used as a gateway to healthy lifestyle. Concurrent use of the two modalities should be investigated further in various populations. Moreover, CAM users in our study indicated to favour support of policy driven decisions related to a healthy lifestyle, consequently, a focus on the Dutch CAM users could work as a gateway to a healthier lifestyle for the general population.

On the other hand, younger age and stress regarding health and the balance between work and family life were found to be positively associated (final multivariable model) with a change into an unhealthy lifestyle. Our data shows that especially younger age was a risk factor for a change into an unhealthier direction. The fact that the young generation seems to be more prone might be due to fact that the restrictions as home confinement during the pandemic has especially impacted their lives by home schooling, working from home and balancing work and childcare (parents) causing a long period of stress resulting into an unhealthier lifestyle. Heightened life stress has been linked to unhealthy eating [35, 36] and stressed people are more likely to crave food high in energy, fats, and sugars [37]. Moreover, parenting is found to be stressful under normative circumstances but pandemic-related data indicate that COVID-19 has led to significant increases in the population’s general stress, a change felt even more acutely for parents than their non-parent counterparts [38]. The results obtained by our study are relevant if we consider that people with stress in relation to balancing work and family care have a 1.7 higher chance of changing into an unhealthy lifestyle than people not perceiving this specific stress.

Some strengths and limitations of this study need to be noted.

Our study has been strengthened by the fact that the survey was conducted during the first critical period of the epidemic in the Netherlands. Responses from over 1000 individuals were rapidly collected within a period of five days from a representative sample of the population. Another strength is that our sample size was sufficiently large for detecting correlations. A limitation of this study is the rather low response rate of 22% to the survey, increasing the risk of non-response bias. Furthermore, because of the urgency to rapidly assess lifestyle changes in a very critical period of the pandemic, the questionnaire was not first piloted among a smaller sample. Although the research team carefully developed and selected life-style related questions and thoroughly discussed comprehensiveness, flow and clarity of the survey, it is not known whether the questionnaire was fully understandable and acceptable for the target population. Another limitation includes the fact that the results are limited by a self-reported questionnaire. The assessment of lifestyle changes was based on individual recall methods, and not by direct measurement of dietary and sleeping pattern, smoking and alcohol consumption. Respondents may thus have either overestimated or underestimated their changes in behaviour. An obvious other limitation of a cross-sectional study design is that it does not allow causal inferences about relationships and thus limits any claim about the directionality of the results. Last, no data on comorbidities (e.g. diabetes, hypertension and obesity) were gathered for the purpose of this study, which might limit the results. Linking with GP data on comorbidities would strengthen future research [15, 16, 31, 32].

The COVID-19 pandemic and following Intelligent lockdown provides an unique window of opportunity to improve lifestyle habits on a population level. This is not only important to combat COVID-19 but also the other pandemic; of obesity and other non-communicable lifestyle-related disease. For a part of the Dutch population, the Corona crisis has already brought a shift in thinking, working and lifestyle behaviour, another large part is now motivated to make such changes. From a public health perspective, it is important to use this unique situation optimally and immediately as this increased motivation is crucial to obtain sustainable lifestyle changes, but may disappear quickly once COVID-19 wanes off. Strategies may include investing in prevention and education (e.g. smoking, drugs, alcohol), health campaigns, lowering taxes on healthy foods and sponsorship of sport facilities. Further studies are warranted to see whether this behavioural change is maintained over time, and how (changing) lifestyle behaviour can affect the susceptibility for and the course of COVID-19. Finally, the results of this study are in line with others showing the potential synergistic relationship between CAM use and healthy lifestyle behaviours [15, 16, 31, 32]. This relation could be targeted in future interventions to increase general wellbeing, symptom control, and clinical outcomes in at-risk populations and might be used as a potential translatable strategy to increase healthy lifestyle behaviours in general populations.

## Supplementary Information

**Additional file 1.**

## Data Availability

The datasets used and/or analysed during the current study are available from the corresponding author on reasonable request.

## Abbreviations

CAM: Complementary and Alternative Medicine
OR: Odds ratio
CI: Confidence interval
