# Supplementary material for: Lifestyle changes during the first wave of the COVID-19 pandemic: a cross-sectional survey in the Netherlands
Source: BMC Public Health. 2021 Jun 25;21:1226. doi: 10.1186/s12889-021-11264-z (PMC8231077; doi:10.1186/s12889-021-11264-z)
Supplement: Supplementary file 1 — Additional file 1. [file 12889_2021_11264_MOESM1_ESM.pdf]

Supplementary material: questionnaire (Dutch)

Lifestyle changes during the first wave of the COVID-19 pandemic: a cross-sectional survey in the Netherlands

Esther T. van der Werf[1,2], Martine Busch[2,3], Miek C. Jong[4,5], H.J. Rogier Hoenders[2,6]

1) Louis Bolk Institute, Bunnik, The Netherlands
2) Dutch Consortium for Integrative Medicine and Health (CIZG)
3) Van Praag Institute, Utrecht, The Netherlands
4) National Research Center in Complementary and Alternative Medicine (NAFKAM), Department of Community Medicine, UiT The Arctic University of Norway, Tromsø, Norway
5) Mid Sweden University, Department of Health Sciences, Sundsvall, Sweden.
6) Center for Integrative Psychiatry (CIP), Lentis Mental Health Care Institution, Groningen, the Netherlands

S01. Wat is uw geboortedatum?

S02. Wat is uw geslacht?

S03. Waar woont u?

- ☐ Postcode:
- ☐ Stad:
- ☐ Gemeente:

S04. In welk type omgeving woont u?

1. Stad of stedelijk
2. Voorstad/buitenwijk
3. Plattelandsgemeente

S05. Wat is uw gezinssituatie?

1. Getrouwd/ samenwonend zonder (thuiswonende) kinderen
2. Getrouwd/ samenwonend met thuiswonende kinderen
3. Alleenstaand zonder (thuiswonende) kinderen
4. Alleenstaand met thuiswonende kinderen
5. Thuiswonend bij (groot)ouder(s)/ familie
6. Studentenhuis/ woongroep

98. Anders, namelijk:

S06. Wat is uw hoogst genoten opleiding? (een opleiding afgerond met diploma of voldoende getuigschrift)

1. Geen opleiding
2. Lagere school
3. LBO, MAVO, VBO, VMBO, LTS, huishoudschool, LEAO, IVO, (M)ULO
4. HAVO, VWO, MBO, HBS, MTS, MEAO
5. HBO
6. WO, postdoctoral

S07. Wat is totale bedrag dat alle leden van uw huishouden gezamenlijk hebben verdiend in het afgelopen jaar?

1. € 0 tot € 9999
2. € 10.000 tot € 24.999
3. € 25.000 tot € 49.999
4. € 50.000 tot € 74.999

5. € 75.000 tot € 99.999
6. € 100.000 tot € 124.999
7. € 125.000 tot € 149.999
8. € 150.000 tot € 174.999
9. € 175.000 tot € 199.999
10. € 200.000 en meer
11. Ik geef liever geen antwoord

Q1.0.

Hartelijk dank dat u deel wilt nemen aan dit onderzoek.

Door middel van dit onderzoek krijgen we graag een beter beeld van uw gebruik van complementaire behandelwijzen en leefstijlveranderingen tijdens de Corona pandemie.

Beantwoord alle vragen naar uw beste weten. Er zijn geen foute of goede antwoorden. Wij zijn geïnteresseerd in uw eigen mening en beoordeling. Uw antwoorden worden anoniem verwerkt en samengevoegd met die van anderen.

Het invullen van deze vragenlijst zal ongeveer 13 minuten van uw tijd in beslag nemen. Selecteer een antwoord voor elke vraag en klik daarna op 'volgende' om verder te gaan. U kunt de vragenlijst op elk moment afsluiten en op een later moment weer openen om af te ronden.

Als u vragen of opmerkingen heeft over de vragenlijst, stuur dan een email naar: healthcare_onderzoek@ipsos-online.com

Q1.1. Heeft u in de afgelopen 3 maanden een of meerdere van de volgende aanbieders bezocht? Geeft u alstublieft alle artsen en andere therapeuten/niet-artsen op die complementaire of alternatieve behandelwijze (CAM) aanbieden en die u hebt bezocht.

U kunt meerdere antwoorden selecteren.

1. Artsen (specialist, huisarts of CAM arts)
2. Homeopaat (artsen/therapeuten/niet-artsen die voornamelijk met homeopathie behandelen)
3. Acupuncturist (artsen/niet-artsen die acupunctuur aanbieden)
4. Natuurgeneeskundige (artsen/niet-artsen die natuurgeneeskunde aanbieden)
5. Antroposofisch zorgverlener (artsen/niet-artsen die antroposofische zorg aanbieden)
6. Osteopaat (artsen/niet-artsen die osteopathie aanbieden)
7. Chiropractor (artsen/niet-artsen die chiropractie aanbieden)
8. Energetisch therapeut
9. (Voet) Reflexoloog
10. Massage therapeut

11. Traditionele Chinese geneeskundige
98. Anders, namelijk (Andere artsen en therapeuten die u met enige vorm van complementaire of alternatieve therapie behandeld hebben, geeft u alstublieft precies op welke methode toegepast werd):
99. Geen van deze

Q1.2. Hoe vaak heeft u in de afgelopen 3 maanden de onderstaande aanbieder(s) bezocht?

Aanbieder(s)	Aantal keer bezocht

Q1.3. Geeft u alstublieft de hoofdreden op, waarom u de laatste keer de onderstaande aanbieder(s) hebt bezocht:

1. Bij een acute ziekte/klacht (met een duur van minder dan 1 maand)
2. Voor de behandeling van een chronische ziekte of de symptomen daarvan (met een duur van meer dan 1 maand)
3. Ter verbetering van het algemeen welbevinden
4. Voor de preventie van Covid-19
5. Voor de behandeling van Covid-19- gerelateerde klachten
6. Anders, namelijk:

Q1.4. Hoe nuttig was het bezoek bij de onderstaande aanbieder(s)?

1. Zeer nuttig
2. Een beetje nuttig
3. Helemaal niet nuttig
4. Ik weet het niet

Q1.5. Heeft u bijwerkingen van de behandeling van de onderstaande aanbieder(s) ervaren?

1. Ja
2. Nee
3. Ik weet het niet

Q1.6. Waar hebt u informatie over de onderstaande aanbieder(s) gekregen?

1. Internet/media
2. Zorgverlener
3. CAM therapeut (niet-artsen)
4. Vrienden/familie etc.
5. Anders, namelijk:

Q2.1.A Geeft u alstublieft **maximaal 3 homeopathische middelen** op, die u in de afgelopen 3 maanden gebruikt hebt.

Geeft u alstublieft zowel de middelen aan die door de arts of de therapeut werden voorgeschreven als ook alle middelen die u op eigen initiatief hebt gebruikt.

Alstublieft het merk (indien bekend) benoemen

1. Bach bloesems:

98. Anders, namelijk:

99. Geen van deze

Q2.1.B Geeft u alstublieft **maximaal 3 kruiden/fytotherapeutische middelen** op, die u in de afgelopen 3 maanden gebruikt hebt.

Geeft u alstublieft zowel de middelen aan die door de arts of de therapeut werden voorgeschreven als ook alle middelen die u op eigen initiatief hebt gebruikt.

Alstublieft het merk (indien bekend) benoemen

1. Echinacea:
2. Passiebloem:
3. Kurkuma:
4. Rode gist rijst:
5. Mariadistel:
6. Ashwagandha (Withania somnifera):
7. Cranberry:
8. Zilverkaars:
9. Ginseng:
10. Ginkgo biloba:

98. Anders, namelijk:

99. Geen van deze

Q2.1.C Geeft u alstublieft **maximaal 3 vitaminen/mineralen** op, die u in de afgelopen 3 maanden gebruikt hebt.

Geeft u alstublieft zowel de middelen aan die door de arts of de therapeut werden voorgeschreven als ook alle middelen die u op eigen initiatief hebt gebruikt.

Alstublieft het merk (indien bekend) benoemen

1. Multivitamine:
2. Hoge dosis vitamin C:
3. Vitamine C (gebruikelijke dosis):
4. Vitamine D:
5. Vitamine B:

6. Selenium/seleen:
7. Zink:
8. Ijzer:
9. Magnesium:
10. Calcium:
98. Anders, namelijk:
99. Geen van deze

Q2.1.D Geeft u alstublieft **maximaal 3 andere CAM producten** op, die u in de afgelopen 3 maanden gebruikt hebt.

Geeft u alstublieft zowel de middelen aan die door de arts of de therapeut werden voorgeschreven als ook alle middelen die u op eigen initiatief hebt gebruikt.

Alstublieft het merk (indien bekend) benoemen

1. Omega 3, 6, 9:
2. Co-enzym Q10:
3. Proteïne/eiwit drank:
4. Probiotics:
5. Glucosamine-chondroitine-MSM:
98. Anders, namelijk:
99. Geen van deze

Q2.2. Geeft u alstublieft de hoofdreden op, waarom u de laatste keer de onderstaande producten hebt gebruikt:

1. Bij een acute ziekte/klacht (met een duur van minder dan 1 maand)
2. Voor de behandeling van een chronische ziekte of de symptomen daarvan (met een duur van meer dan 1 maand)
3. Ter verbetering van het algemeen welbevinden
4. Voor de preventie van Covid-19
5. Voor de behandeling van Covid-19- gerelateerde klachten
6. Anders, namelijk:

Q2.3. Hoe nuttig vond u de onderstaande producten?

1. Zeer nuttig
2. Een beetje nuttig
3. Helemaal niet nuttig
4. Ik weet het niet

Q2.4. Heeft u bijwerkingen van de behandeling met de onderstaande producten ervaren?

1. Ja
2. Nee
3. Ik weet het niet

Q2.5. Waar hebt u informatie over de onderstaande producten gekregen?

1. Internet/media
2. Zorgverlener
3. CAM therapeut (niet-artsen)
4. Vrienden/familie etc.
5. Anders, namelijk

Q3.1. Heeft u in de afgelopen 3 maanden een of meerdere van de volgende zelfzorg methoden beoefend?

1. Meditatie/Mindfulness
2. Yoga
3. Qigong
4. Tai Chi
5. Ontspanningstechnieken
6. Visualisatie
7. Bidden voor de eigen gezondheid
8. Schilderen/musiceren voor de eigen gezondheid

98. Anders, namelijk:
99. Geen van deze

Q3.2. Hoe vaak heeft u in de afgelopen 3 maanden de onderstaande zelfzorg methode(n) beoefend?

1. Dagelijks
2. Wekelijks
3. Maandelijks
4. Minder dan 1 keer per maand

Q3.3. Geeft u alstublieft de hoofdreden op, waarom u de laatste keer de onderstaande zelfzorg methode(n) hebt beoefend?

1. Bij een acute ziekte/klacht (met een duur van minder dan 1 maand)
2. Voor de behandeling van een chronische ziekte of de symptomen daarvan (met een duur van meer dan 1 maand)
3. Ter verbetering van het algemeen welbevinden

4. Voor de preventie van Covid-19
5. Voor de behandeling van Covid-19- gerelateerde klachten
6. Anders, namelijk:

Q3.4. Hoe nuttig vond u de onderstaande zelfzorg methode(n)?

1. Zeer nuttig
2. Een beetje nuttig
3. Helemaal niet nuttig
4. Ik weet het niet

Q3.5. Heeft u bijwerkingen of complicaties van de onderstaande zelfzorgmethode(n) ervaren?

1. Ja
2. Nee
3. Ik weet het niet

Q3.6. Waar hebt u informatie over de onderstaande zelfzorg methode(n) gekregen?

1. Internet/media
2. Zorgverlener
3. CAM therapeut (niet-artsen)
4. Vrienden/familie etc.
5. Anders, namelijk:

Q4.1.A Geeft u alstublieft antwoord op de onderstaande vragen op een schaal van 1 (= helemaal niet) tot 5 (= heel erg).

1. Helemaal niet
2. Niet
3. Neutraal
4. Erg
5. Heel erg

1. Hoe bezorgd/bang bent u om besmet te raken met Covid-19?
2. Hoe bezorgd/bang bent u dat een van uw naasten (familie/vrienden) besmet zal raken met Covid-19?

Q4.1.B Denkt u dat het Covid-19 gevaarlijker is dan de normale (seizoens) griep?

1. Veel minder gevaarlijk

2. Minder gevaarlijk
3. Even gevaarlijk
4. Gevaarlijker
5. Veel gevaarlijker

Q4.2. Hoeveel glazen alcohol drinkt u gemiddeld per week?

Q4.3. Rookt u? Zo ja, hoeveel sigaretten per dag:

1. Ja, aantal sigaretten per dag:
2. Nee

Q4.4. Hoeveel dagen gemiddeld per week beweegt u minstens 30 minuten per dag extra op de gewone beweging in het dagelijks leven (bijv. sporten, wandelen, fietsen)?

Aantal dagen:

Q4.5. Op een schaal van 0 (heel slecht) tot 10 (uitstekend) beoordeel ik mijn slaap gemiddeld met het cijfer:

1. Heel slecht
2.
3.
4.
5.
6.
7.
8.
9.
10. Uitstekend

Q4.6. Geeft u alstublieft voor de onderstaande factoren aan in hoeverre u **sinds de Corona crisis** (vanaf maart 2020 tot nu) hier meer of minder gebruik van maakt.

1. Meer
2. Even veel
3. Minder

99. Weet niet

1. Het drinken van alcohol.
2. Het roken van sigaretten.
3. Extra bewegen bovenop de gewone dagelijkse beweging (bijv. sporten, wandelen, fietsen).
4. Het aantal uren slaap.

Q4.7. Geeft u alstublieft voor de onderstaande factoren aan in hoeverre u **sinds de Corona crisis** (vanaf maart 2020 tot nu) er meer of minder stress door ervaart.

1. Meer stress
2. Even veel stress
3. Minder stress

99. Weet niet / niet van toepassing

1. Stress met betrekking tot werk.
2. Stress met betrekking tot gezondheid.
3. Stress met betrekking tot sociale contacten.
4. Stress met betrekking tot verdeling werk en zorg voor kinderen.
5. Stress met betrekking tot mantelzorg.
6. Stress met betrekking tot inkomen en financiën.
7. Stress met betrekking tot de toekomst.
8. Stress met betrekking tot anders, namelijk:

Q4.8. Eet u meer of minder van de onderstaande soorten voedsel **sinds de Corona crisis** (vanaf maart 2020 tot nu)?

1. Meer
2. Even
3. Minder
4. Weet niet

1. Fruit per dag

2. Groente per dag
3. snacks (koek/snoep/taart/chips/) per dag

Q4.9 In hoeverre bent u **sinds de Corona crisis** (vanaf maart 2020 tot nu) bewuster bezig bent met uw voedingspatroon dan ervoor.

1. Meer bewust van mijn voedingspatroon
2. Even bewust van mijn voedingspatroon
3. Minder bewust van mijn voedingspatroon
4. Weet niet

Q5.0. De volgende vragen gaan over uw leefstijl. Wanneer wij het over leefstijl hebben, bedoelen wij zes aspecten die samen van grote invloed zijn op de lichamelijke en geestelijke gezondheid van de mens (voeding, beweging, slaap, sociaal, ontspanning en zingeving).

Q5.1. Bent u in het algemeen tevreden met uw leefstijl?

1. Ja
2. Nee
3. Weet niet

Q5.2. Denkt u dat een verandering van leefstijl uw risico op het krijgen van het Corona virus (COVID-19) kan beïnvloeden?

1. Ja
2. Nee
3. Weet niet

Q5.3. Denkt u dat een verandering van leefstijl het ziektebeloop van het Corona virus (COVID-19) kan beïnvloeden bij een eventuele besmetting?

1. Ja
2. Nee
3. Weet niet

Q5.4. Beïnvloedt de Corona crisis uw leefstijl?

1. Nee
2. Ja, ik leef **ongezonder**
3. Ja, ik leef **gezonder**

Q5.5. Ik leef **gezonder** door de Corona crisis omdat ik:

U kunt meerdere antwoorden selecteren

1. Meer fruit en groente eet
2. Meer beweeg
3. Meer ontspan
4. Meer sociaal contact aanga
5. Meer bezig ben met zingeving

Q5.6. Op welke van de onderstaande gebieden zou u uw leefstijl blijvend willen veranderen na de Corona crisis?

U kunt meerdere antwoorden selecteren.

1. Gezonde voeding
2. Afkomen van een verslaving (alcohol, roken, drugs, seks, suiker, Netflix/tv, gamen)
3. Bewegen
4. Slaap
5. Stress
6. Sociale contacten
7. Zingeving

98. Anders, namelijk:
99. Ik wil mijn leefstijl niet aanpassen

Q5.7. Stel dat u uw leefstijl blijvend wilt veranderen. Wat is er nodig voor u om een verandering in uw leefstijl in te zetten en vol te houden, ook na de Corona crisis?

U kunt meerdere antwoorden selecteren.

1. Vrije keuze en vergoeding basispakket verzekering van behandelaar op het gebied van leefstijl en complementaire zorg;
2. Begeleiding vanuit huisarts/gezondheidscentrum/wijkteam;
3. Online advies & begeleiding;
4. Gezond keuzenaanbod op het werk/opleiding (eten, drinken, beweegaanbod in de pauze zoals fitness, yoga, Tai Chi, mindfulness, muziek etc.);
5. Betaalbare en makkelijk toegankelijke gezonde voeding

98. Anders, namelijk:
99. Geen van deze

Q6.1.

U bent aan het einde van de vragenlijst gekomen. Bedankt voor uw medewerking en uw tijd.
